# Prospective collection of blood plasma samples to identify potential biomarkers for the prehospital stroke diagnosis (ProGrEss-Bio): study protocol for a multicenter prospective observational study

**DOI:** 10.3389/fneur.2023.1201130

**Published:** 2023-07-06

**Authors:** Frederik Geisler, Lisa Haacke, Maren Lorenz, Eugen Schwabauer, Matthias Wendt, Lydia Bernhardt, Eman Dashti, Erik Freitag, Alexander Kunz, Christina Hofmann-Shen, Martina Zuber, Carolin Waldschmidt, Farid I. Kandil, Kai Kappert, Chantip Dang-Heine, Irina Lorenz-Meyer, Heinrich J. Audebert, Joachim E. Weber

**Affiliations:** ^1^Department of Neurology, Charité - Universitätsmedizin Berlin, Corporate Member of Freie Universität Berlin and Humboldt-Universität zu Berlin, Berlin, Germany; ^2^Department of Neurology, Vivantes Klinikum Neukölln, Berlin, Germany; ^3^Department of Neurology, Unfallkrankenhaus Berlin, Berlin, Germany; ^4^Department of Neurology, Klinikum Ernst von Bergmann, Potsdam, Germany; ^5^Kliniken Beelitz, Teaching Hospital of Brandenburg Medical School Theodor Fontane, Beelitz-Heilstätten, Germany; ^6^Department of Neurology, Vivantes Humboldt-Klinikum, Berlin, Germany; ^7^Department of Nephrology and Medical Intensive Care, Charité - Universitätsmedizin Berlin, Corporate Member of Freie Universität Berlin and Humboldt-Universität zu Berlin, Berlin, Germany; ^8^Institute of Social Medicine, Epidemiology and Health Economics, Charité - Universitätsmedizin Berlin, Corporate Member of Freie Universität Berlin and Humboldt-Universität zu Berlin, Berlin, Germany; ^9^Institute of Diagnostic Laboratory Medicine, Clinical Chemistry and Pathobiochemistry, Charité – Universitätsmedizin Berlin, Corporate Member of Freie Universität Berlin and Humboldt-Universität zu Berlin, Berlin, Germany; ^10^Berlin Institute of Health at Charité – Universitätsmedizin Berlin, Berlin, Germany; ^11^Center for Stroke Research Berlin, Charité - Universitätsmedizin Berlin, Corporate Member of Freie Universität Berlin and Humboldt-Universität zu Berlin, Berlin, Germany; ^12^German Centre for Cardiovascular Research (DZHK), Partner Site Berlin, Berlin, Germany

**Keywords:** cerebrovascular disease, stroke, intracerebral hemorrhage, prehospital emergency medicine, emergency medicine, mobile stroke units, biomarkers, stroke mimics

## Abstract

**Introduction:**

Intravenous thrombolysis (IVT) and mechanical thrombectomy (MT) are well-established, evidence-based, time-critical therapies that reduce morbidity and mortality in acute ischemic stroke (AIS) patients. The exclusion of intracerebral hemorrhage (ICH) is mandatory and has been performed by cerebral imaging to date. Mobile stroke units (MSUs) have been shown to improve functional outcomes by bringing cerebral imaging and IVT directly to the patient, but they have limited coverage. Blood biomarkers clearly distinguishing between AIS, ICH, and stroke mimics (SM) could provide an alternative to cerebral imaging if concentration changes are detectable in the hyperacute phase after stroke with high diagnostic accuracy. In this study, we will take blood samples in a prehospital setting to evaluate potential biomarkers. The study was registered in the German Clinical Trials Register (https://drks.de/search/de) with the identifier DRKS00023063.

**Methods and analysis:**

We plan a prospective, observational study involving 300 patients with suspected stroke and symptom onset of ≤4.5 h before the collection of biomarkers. Study participants will be recruited from three sites in Berlin, Germany during MSU deployments. The focus of the study is the collection of blood samples from participants at the prehospital scene and from participants with AIS or ICH at a second-time point. All samples will be analyzed using targeted and untargeted analytical approaches. Study-related information about participants, including medical information and discharge diagnoses from the subsequent treating hospital, will be collected and documented in an electronic case report form (eCRF).

**Discussion:**

This study will evaluate whether a single blood biomarker or a combination of biomarkers can distinguish patients with AIS and ICH from patients with stroke and SM in the early phase after symptom onset in the prehospital setting. In addition, the kinetics of blood biomarkers in AIS and ICH patients will be investigated. Our goal is to evaluate new ways to reliably diagnose stroke in the prehospital setting and thus accelerate the application of evidence-based therapies to stroke patients.

## Introduction

Stroke is a major cause of mortality and disability worldwide (1). Cerebral imaging and clinical examination play a crucial role, and blood biomarkers may be useful in diagnosing and evaluating treatment options in stroke patients (2–4). Intravenous thrombolysis (IVT) and mechanical thrombectomy (MT) alone or in combination are established evidence-based revascularization treatments for acute ischemic stroke (AIS) patients (5, 6). However, the effectiveness of these therapies is highly time dependent (7), and the exclusion of certain contraindications before IVT is mandatory (8, 9). To date, intracerebral hemorrhage (ICH), one of the most important contraindications, is only reliably detectable by cerebral imaging, either with computed tomography (CT) or magnetic resonance imaging (MRI), which is thus mandatory before the initiation of IVT (10).

Mobile stroke units (MSUs)—ambulances with a CT scanner and a point-of-care (POC) laboratory on board—significantly reduce the alarm-to-treatment time (ATT) compared to regular care and increase the number of patients treated within 60 min after the onset of symptoms (11–14), two large studies (Berlin_PRehospital Or Usual Delivery of Acute Stroke Care (B_PROUD) and BEnefits of Stroke Treatment Delivered Using a Mobile Stroke Unit [BEST-MSU]) independently demonstrated that treatment on board an MSU led to a significant reduction in disability at 90 days (15, 16). Furthermore, in cases of large vessel occlusion (LVO), patients can be selectively transported to the next appropriate hospital offering mechanical thrombectomy.

The number of operational MSUs is increasing around the globe (Map of active MSUs: https://www.prestomsu.org/); however, maintenance and operational costs make them particularly suitable for use in urban areas (17, 18). Therefore, there is a substantial unmet need to explore new ways of diagnosing AIS, ICH, or diseases that mimic stroke [so-called stroke mimics (SMs)] without cerebral imaging, thereby reducing treatment times even outside the range of action of MSUs in the prehospital setting.

Identification of potential blood-based biomarkers may be a first step for the subsequent use of POC diagnostics in regular ambulances to distinguish AIS from ICH and SMs, initiate IVT in the prehospital setting, and improve patient allocation to specialized centers (19). In recent years, several blood-based biomarkers have shown promise for differentiating between AIS and ICH. These biomarkers include RBP-4, NT-proBNP, GFAP, FXII, plasminogen, and prolactin (20–22). Additionally, biomarkers such as S100B, MMP-9, and CRP (23) have been evaluated for their ability to distinguish between stroke and other acute neurological conditions in the early in-hospital phase (24–27). However, to date, no single blood biomarker or panel of biomarkers has been identified to replace cerebral imaging (28). Moreover, to the best of our knowledge, there are almost no studies on blood-based biomarkers during the very early prehospital phase or analyzing the kinetics shortly after the onset of symptoms (29). However, one study showed promising sensitivity and specificity regarding the kinetics of prehospital measured GFAP in differentiating between AIS and ICH (30).

Blood biomarkers must not only confirm the clinical diagnosis of AIS and rule out ICH with a high degree of certainty but also detect AIS and ICH very early after the onset of symptoms to start causal therapies as soon as possible. Most studies have investigated blood biomarkers only in the later in-hospital phase. Therefore, in this study, we plan a multicenter prospective observational study by drawing blood from patients who are treated in the prehospital field by an MSU neurologist and want to identify biomarkers suitable for the differentiation between AIS, ICH, and SMs.

## Methods and analysis

The manuscript was written according to the SPIRIT 2013 guidelines for protocols and clinical trials (31).

### Study title

The name of the study, ProGrEss-Bio, is the translation of the German title “**Pro**spektive **G**ewinnung von Blutplasma-P**r**oben zur **E**rfa**ss**ung geeigneter **Bio**marker für die prähospitale Schlaganfallforschung.”

### Hypotheses

#### Primary hypothesis

One or a group of different blood biomarkers enables the differentiation between AIS and ICH in patients with acute stroke with sufficient sensitivity, specificity, and predictive values.

#### Secondary hypothesis

One or a group of different blood biomarkers enables the differentiation between acute stroke and SM in patients with suspected stroke with sufficient sensitivity, specificity, and predictive values. The kinetics of biomarkers in the early phase of a stroke may improve discriminatory power.

### Study design

**Figure 1:**
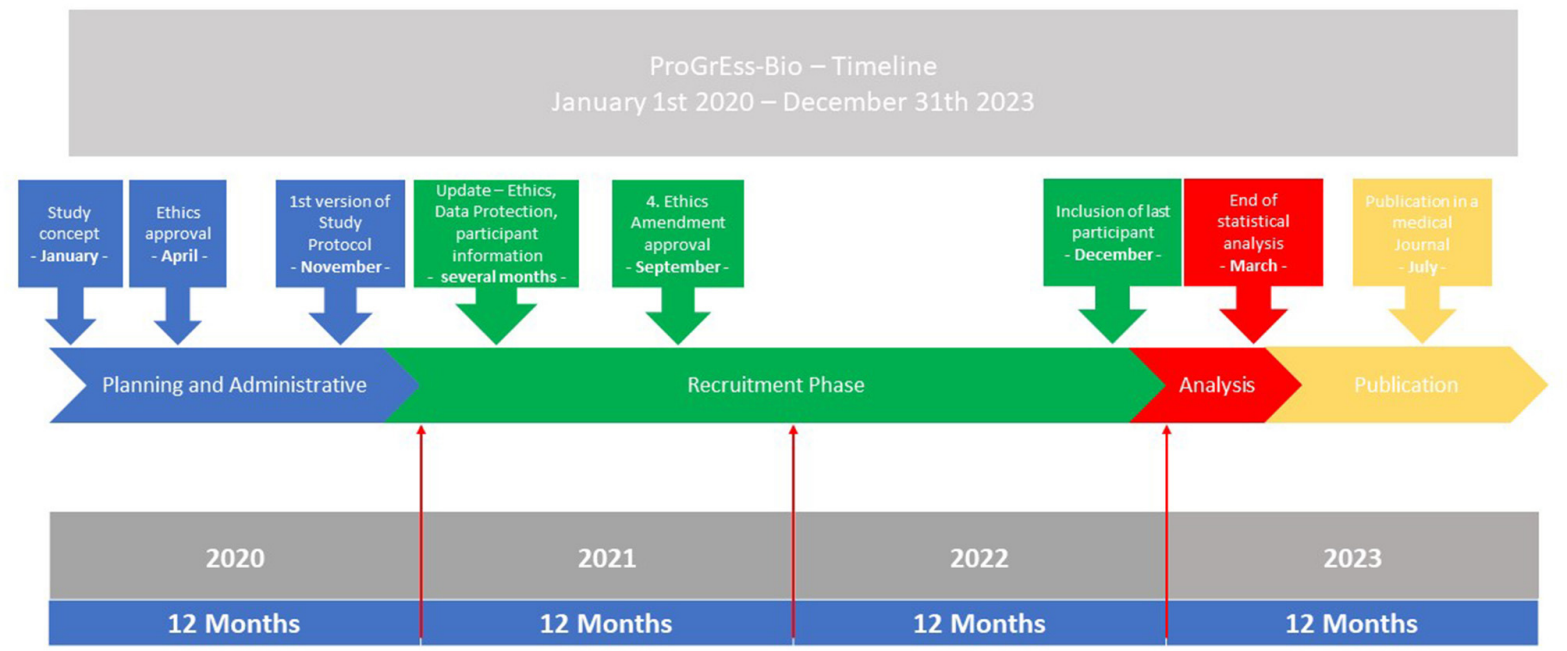
Timeline. The timeline for the study from 2020 to 2023 is shown. The parts of the planning, administrative, and recruitment phases are depicted in the blue and green boxes. The analysis and publication phases are found in the red and yellow box. At the bottom of the graphic, the months and years are shown.

**Figure 2:**
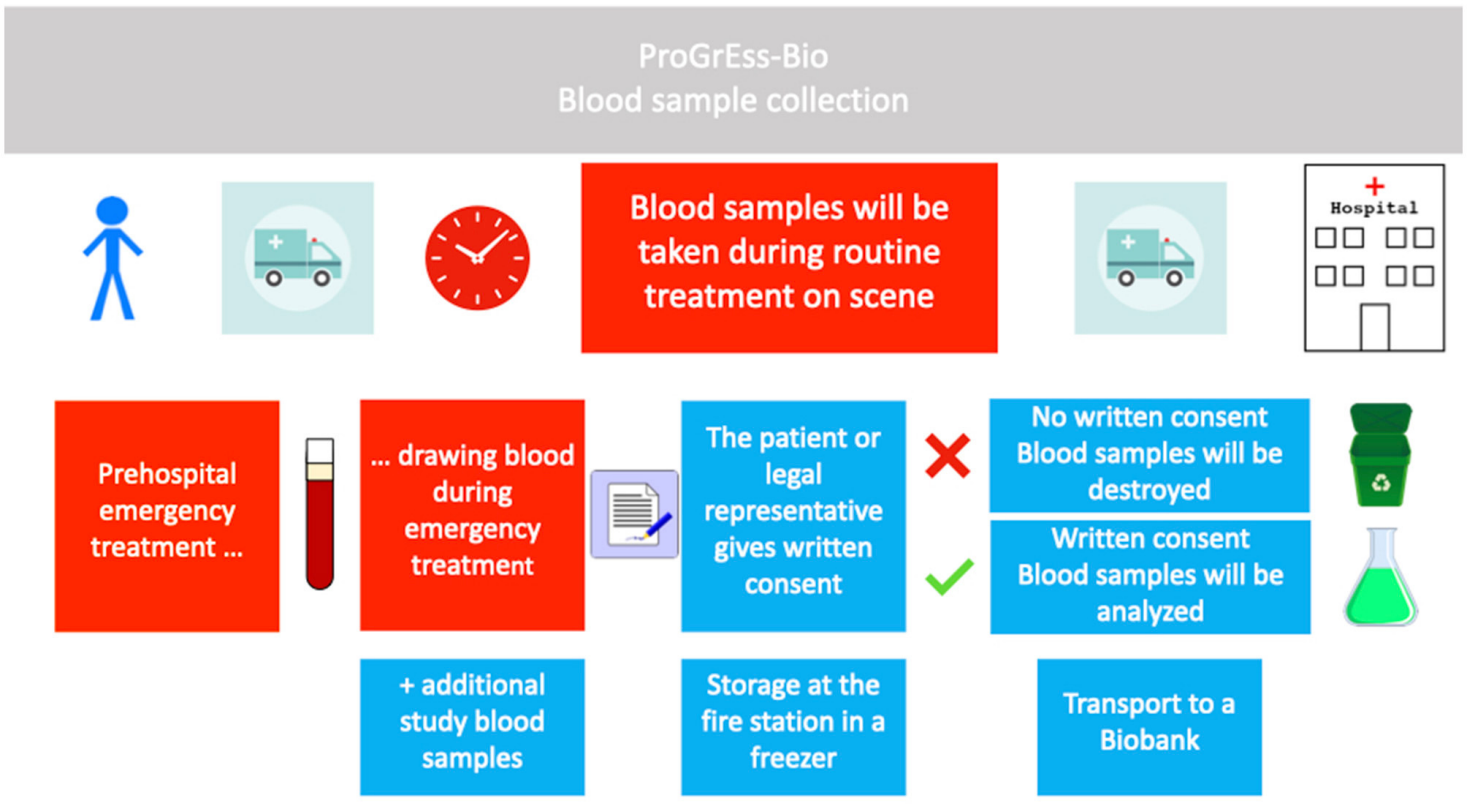
Conduction of the study. Procedure for including patients in the study is shown in chronological order from left to right. Boxes in red describe procedures during routine emergency treatment, and boxes in blue describe study-specific procedures. Blood samples will only be analyzed in cases where the patient or legal representative gives written informed consent. The blood sample is temporarily stored in the fire or rescue station and later in a biobank.

ProGrEss-Bio (“prospective collection of blood plasma samples to identify potential biomarkers for the prehospital stroke diagnosis”) is a multicenter, prospective observational study that aims to investigate patients with suspected stroke within 4.5 h of the onset of symptoms. The study's primary objective is to identify blood-based biomarkers that can help in identifying stroke subtypes and differentiating between stroke and other acute neurological conditions imitating stroke, i.e. SMs. Details for the timeline can be found in Figure 1. The study is being conducted at three MSU study platforms (also known as Stroke Emergency Mobiles [STEMO]) of the Berlin Fire Department. Patients suspected of possibly having an acute stroke or SM based on the initial assessment of the MSU neurologist are recruited during routine prehospital care by the MSU neurologist. Details can be found in Figure 2.

In contrast to many other studies, we plan to use a broad spectrum of potential biomarkers using a so-called “multi-omics” approach [proteins (proteomics), genes (genomics), ribonucleic acid (RNA [transcriptomics]), and metabolites (metabolomics)]. A second study-related blood sample will be collected from AIS and ICH patients 20 to 60 min after the collection of the first sample, usually done upon the patient's arrival at the hospital, the study allows for the examination of concentration changes and kinetics in the early phase. We plan to include 300 patients in this study. The study will be initially implemented at one MSU (STEMO 3600) and then rolled out to the other two MSUs. Universal trial number (UTN): U1111–1258-2477.

### Study population

#### Inclusion criteria

Participants are eligible for the study if they meet the following inclusion criteria:

- Age ≥ 18 years.
- Suspected or possible stroke or SM based on the initial assessment of the treating MSU neurologist.
- Symptom onset of ≤4.5 h before the collection of biomarkers.
- Written informed consent will be obtained from the participant or their legal representative. Informed consent can be given up to 12 weeks after the collection of the biomarkers, but before further analyses, either by the patient or their legal representative.

#### Exclusion criteria

Participants who meet any of the following criteria are excluded from study entry:

- Drawing blood is not possible, either for technical or medical reasons.
- Withdrawal of written consent for study participation by a participant or their legal representative. No informed consent is provided up to 12 weeks after drawing blood because, by then, blood samples will be destroyed.

The symptom onset refers to the information the MSU neurologist has at the scene, as it best reflects the real situation in the case of a possible IVT decision.

In cases of severe medical events in the days or weeks before drawing blood, e.g., myocardial infarction, the study physician can elaborate on these events in detail in an open field of the electronic case report form (eCRF). These events possibly affect the measurement of the blood biomarkers and are, therefore, usually recorded. Further pre-existing conditions, such as arterial hypertension, are mandatory fields in the eCRF.

### Outcomes

The main objective of this study is to discover blood-based biomarkers suitable for acute stroke therapies without cerebral imaging and thus potentially improve prehospital diagnosis. These biomarkers need to reliably detect strokes and distinguish between AIS and ICH. In addition, they should be rapidly measurable and readily available in as many regular ambulances as possible worldwide. Concentration changes of potential blood biomarkers must be reliably detectable very early after symptom onset in POC devices to start IVT as early as possible in the prehospital setting. In many studies, blood is drawn in the later phase of AIS; therefore, early concentration changes of these biomarkers are unknown. Additionally, we do not plan to only collect blood samples very early but plan to use novel proteomics, transcriptomics, and metabolomics approaches and thus investigate many biomarkers simultaneously. In some patients, statements about the kinetics will be possible since two blood samples at different time points are collected.

#### Primary and secondary targets

As this is an explorative study (discovery phase), the primary target is to identify a single blood-based biomarker, or a panel of biomarkers, with strong differentiation between the AIS and the ICH groups, i.e., to identify candidates for a prediction model that will then be tested and refined in a subsequent study.

The same is true for the secondary target, namely, to identify a single blood-based biomarker or, again, a group of markers with great differences between stroke and SM patients.

To this end, the absolute values of blood biomarkers and their kinetics in AIS and ICH patients will be determined. We expect a change in the concentration of biomarkers over time in the acute phase after AIS and ICH patients due to vascular injury and subsequent neuronal cell injury, whereas we expect no such changes in SM patients.

### Biomarkers

Out of the proteomics and metabolomics data, we plan to identify targets for the differentiation between AIS and ICH and between stroke and SM. Furthermore, we will measure single blood biomarkers previously tested and considered promising candidates for the differentiation between AIS, ICH, and SM. Therefore, we will measure apo CIII, copeptin, D-Dimer, endostatin, EGFR, eotaxin, FXII, GFAP, HSC-70, MMP inhibitor-4, MMP-9, prolactin, NT-proBNP, plasminogen, RBP-4, S100A12, S100-B, and UCHL-1 (20–23, 32–35).

### Collection and processing of data and samples

#### Blood sampling and processing

The first study-related blood sample will be taken after all steps of the necessary emergency treatment are completed. A second study-related blood sample in AIS or ICH will be drawn between 20 and 60 min subsequently, if possible, i.e., the patient will not be transported by regular ambulance alone or for other reasons, e.g., life-saving therapies are required, making the collection of a second blood sample impossible.

**Figure 3:**
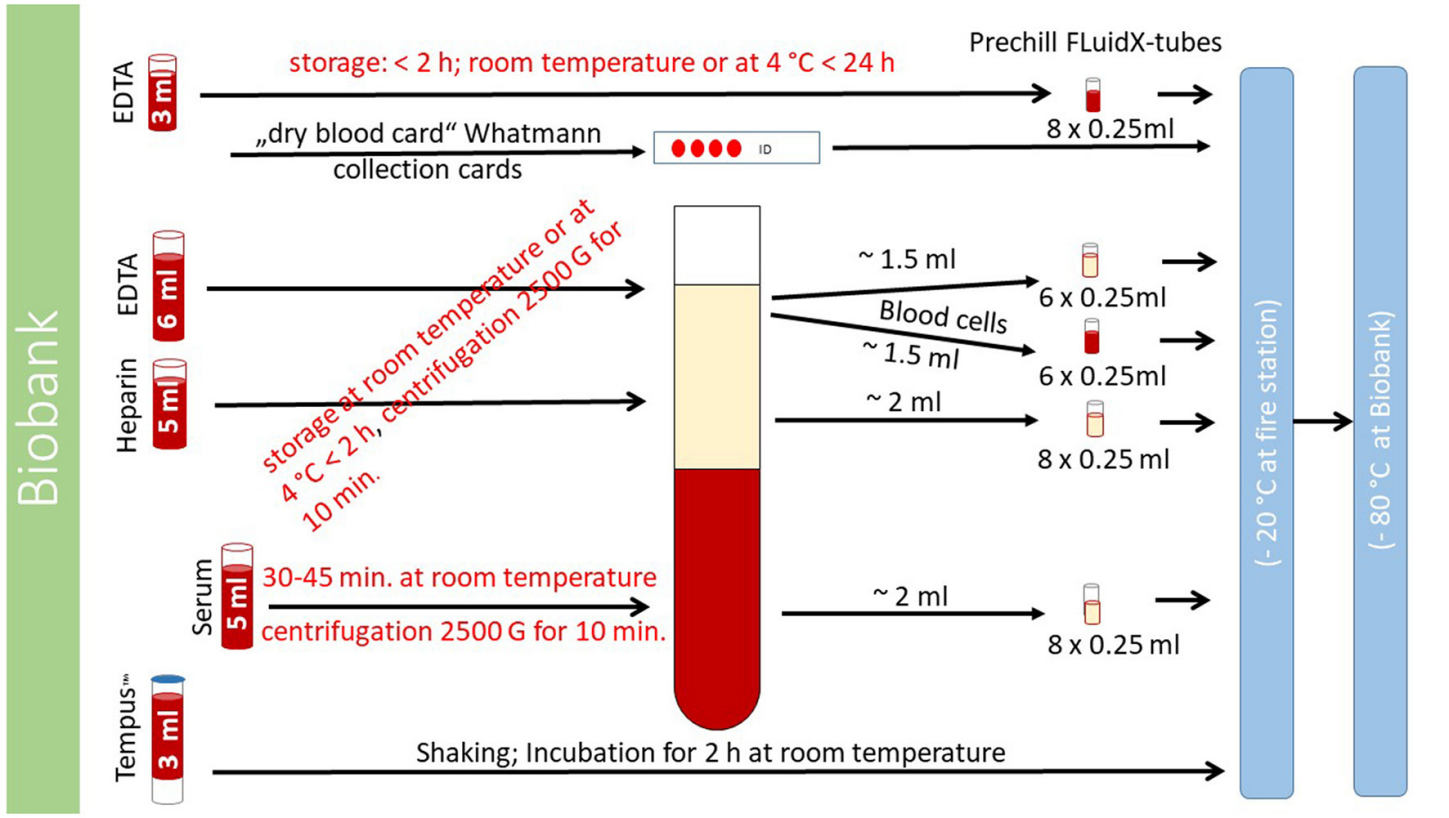
Handling of blood samples. The process of handling blood samples is described here. Details of the exact procedure can be found in the text “collection and processing of blood samples.”

All tubes for each study participant will be individually pre-labeled, pre-coded, and packed with a unique identifier (ID). Identification of the study participants will only be possible with this unique ID. The following steps are shown in detail in Figure 3. Two ethylenediaminetetraacetic acid (EDTA) tubes (6 and 3 ml), one heparin tube (5 ml), one serum tube (5 ml) (VACUETTE^®^, blood drawing tubes, from Greiner Bio-One, Austria, Kremsmünster), a Tempus™-tube (3 ml) [TEMPUS™, blood ribonucleic acid (RNA) tube, Applied Biosystems™ by Thermo Fisher Inc., United States, Waltham, MA], and a small amount of blood in a full blood tube for international normalized ratio measurement and dry blood cards will be used for blood sampling in study participants. The serum tube will be collected first, and the Tempus™-tube will be the last.

One EDTA tube (6 ml), the heparin tube (5 ml), and the serum tube (5 ml) will be centrifuged with 2,500 × g in a centrifuge (CompactStar CS4, maximum rotation speed 6500 min.^−1^/4000 × g; maximum capacity for 6 tubes; from Avantor™ delivered by vwr™, United States, Radnor, PA) aboard the MSU. After centrifugation, blood cells will be separated from blood plasma. The blood cells and blood plasma originating from the EDTA tube will be divided into 2 × 6 different samples (0.25 ml volume for each sample; six samples for blood cells and six samples for blood plasma) by pipetting them into small “FluidX-tubes.” The blood plasma originating from the heparin and serum tubes will be divided into 8 different samples each (0.25 ml volume for each sample) by pipetting them into different “FluidX-tubes.” The non-centrifugated EDTA tube (3 ml) will be divided into 8 samples of 0.25 ml each and pipetted into “FluidX-tubes.”

The Tempus™ tube (3 ml) will be shaken, non-centrifugated, incubated for 2 h at room temperature, and then stored in a freezer (at −20°C). One drop of full blood is placed on a dry blood card (Whatmann collection cards).

The 36 “FluidX-tubes” can be stored for up to 2 h in a refrigerator (at 4°C) aboard the MSU. The 37 tubes (36 “FluidX tubes” and one Tempus™ tube) and blood card will be transported with the MSU to the fire or rescue station and then stored in a locked freezer (at −20°C) in a secured room. These samples will be transported once a month or in shorter intervals, if necessary, to the fully certified biobank of the Max-Delbrück-Centrum (MDC) and stored there at −80°C. For details on processing, see Figure 3.

#### Data storage and management

Data management will be conducted by the data management team of the clinical study center (CSC) of the Charité – Universitätsmedizin Berlin and the Center for Stroke Research Berlin (CSB). The clinical data of each patient will be pseudonymized using a unique identification number (pseudonym) created and assigned by the data trust center of the Charité (CHA-THS). Identifying data will be stored separately from the pseudonymized clinical data at the respective MSU platforms. Identifying data can only be accessed by the study teams of the respective MSUs according to a predefined concept of access rights and rules. Pseudonymized study-related data can be accessed by the study teams of the respective MSUs as well as the leading scientists and the biostatistician according to a predefined concept of access rights and rules. A statistical analysis will be conducted based on pseudonymized data.

ProGrEss-Bio uses an open-source electronic data entry and data management system (REDCap). Pseudonymized data will be collected using a web-based central eCRF on a tablet. Identifying data will be deleted after the completion of the study and will be stored for 10 years within the CHA-THS before being deleted, allowing no depseudonymization of the results.

#### Primary analysis

The prehospital distinction between patients with AIS and ICH without cerebral imaging and solely based on a single or a combination of blood biomarkers.

#### Secondary analysis

The prehospital distinction between patients with a stroke and SM without cerebral imaging and solely based on one or a combination of blood biomarkers.

#### Tertiary analysis

Determination of the kinetics of blood biomarkers within the acute phase of a stroke (≤4.5 h after symptom onset) by drawing blood directly after MSU arrival at the scene and 20–60 min later to detect and observe early changes in the concentrations of blood biomarkers.

### Definitions

The main diagnosis of the hospital discharge letter will be used to decide whether the patient suffered from an AIS, an ICH, or an SM. In cases of more than one diagnosis, the most likely differential diagnosis was used. In cases where no clear distinction between AIS, ICH, and SM was possible, patients were excluded from the analysis. TIA patients will be considered to be part of the AIS group, but how many patients suffered from a TIA and AIS will be reported.

Stroke entities or SMs in the literature are usually defined by the following definitions:

- Stroke:

- Ischemic stroke: An episode of neurological dysfunction caused by focal cerebral, spinal, or retinal infarction but infarction caused by the brain, spinal cord, or retinal cell death is attributable to ischemia, based on pathological imaging or clinical evidence of cerebral, spinal cord, or retinal focal ischemic injury based on symptoms persisting ≥24 h or until death and other excluded etiologies (36).
- Intracerebral hemorrhage: Rapidly developing clinical signs of neurological dysfunction attributable to a focal collection of blood within the brain parenchyma or the ventricular system that is not caused by trauma (36).

Further details on the diagnosis of stroke can be found elsewhere (36, 37).

- Stroke mimics:

- Certain diseases such as syncope, epileptic seizures, hypoglycemia, hyperglycemia, migraine aura, dissociative disorder, and so on. A stroke is initially clinically suspected, but the diagnostic criteria for stroke are not fulfilled (36).

#### Sample size

In this explorative study (“discovery phase”), no decisive sample size calculation can be performed, as the effect size of the parameters with respect to predicting to which group the patients belong will become clear only through and at the end of this study. Thus, the envisaged sample sizes will be used to determine the effects that can be detected with them (see next section).

Since, for all patients, the ultimate diagnosis (AIS, ICH, or SM) is known only in retrospect, patients will be included until the targeted sample sizes of all groups are completed or the anticipated study end is reached.

### Statistical analysis

For the primary target, out of all available proteomics and metabolomics data, possible predictors for the differentiation between AIS and ICH are extracted by means of logistic regression. Similar to the selection process for the secondary target, predictors will be chosen to differentiate between AIS (and ICH) on the one side and SM on the other side.

For both targets of this “discovery phase,” all predictors will be tested individually to assess their prediction quality (Hosmer). Predictors with a “significant contribution” (that is, a *p*-value in the Chi-squared test with *p* < α = 0.20) will be acknowledged as suitable candidates and further investigated in the combined Phases 2 and 3 (“Derivation Phase” and “Validation Cohort”) taking place at a later phase of the biomarker project with a new sample.

Additionally, sensitivity, specificity, and predictive values will be tested for potential biomarkers.

### Power analysis

For the primary target, the data from 80 AIS and 80 ICH patients will be used. Given the elevated alpha = 0.20 used here, the standard value of beta = 20% (corresponding to a power of 80%), and the use of the Chi-squared test, the sample will be sufficient to detect all large, medium, and small effects of w ≥ 0.17 (Cohen's d ≥ 0.30). For the secondary target, for which data from 40 AIS (taken as a random sample out of the pool of 80 patients) and 40 SM patients will be used, the numbers will suffice to detect large, medium, and some smaller effects with w ≥ 0.24 (Cohen's d ≥ 0.40) and thus all effects with a minimally clinically important difference (MID).

## Discussion

In our study, the first important research goal is to identify one or a series of blood biomarkers that may allow differentiation between AIS and ICH and will be validated in later and larger phases of this study. Suitable biomarkers will then be investigated by us and possibly in animal models by other laboratories regarding their discriminatory power between AIS, ICH, and SMs.

In a small study sample of 74 patients assessed on MSU at the scene, the blood biomarker glial fibrillary acidic protein (GFAP) was elevated with a cut-off value above 0,29 ng/ml exclusively in ICH patients, but, in particular, patients with smaller intracranial bleedings often presented with values below the mentioned cut-off value (38). In a review that included four studies with data from 340 patients, GFAP blood levels were significantly elevated in ICH compared to AIS patients, while in both stroke subtypes, the GFAP concentrations correlated with stroke severity (39). In a prospective study with 251 patients, GFAP and ubiquitin carboxy-terminal hydrolase-L1 (UCH-L1) were measured and showed higher ICH levels than the AIS patients (32). GFAP showed higher diagnostic accuracy with 20 times higher values in ICH patients and, depending on the cut-off values, a sensitivity of 75% and a specificity of 84% but was not able to differentiate with acceptable certainty between AIS and ICH patients (32, 40). Other authors also reported a correlation of GFAP with clinical severity and higher concentrations in ICH compared to AIS and non-stroke patients (33, 41, 42). However, GFAP levels may be normal or only slightly elevated in patients with small intracerebral hemorrhages. Therefore, this blood biomarker alone appears not to be sufficient to decide whether to treat a suspected AIS patient with IVT without prior imaging results.

A broader proteomics approach was used in an exploratory pilot study while identifying nine candidate biomarkers in a panel of 495 protein groups but only three AIS patients, four ICH patients, and two healthy control persons (21). Another approach was proposed with the measurement of certain microRNAs that are relatively stable and may allow the identification of AIS patients, especially in the ultra-early phase after an ischemic stroke, as well as circulating tRNA fragments (43–45). Additionally, several metabolites, often called metabolomics, may help identify AIS patients, but further research is required (46, 47). These new approaches are an emerging field, but to date, only small or pilot studies have been published, and their results are not yet suited for clinical use.

The second important objective of our study is to distinguish stroke patients from SM patients.

The differentiation between AIS, ICH, and SM patients was reported in a study with 1,308 participants based on the blood biomarkers NT-proBNP and endostatin combined with other variables, yielding a positive predictive value of 80.8%, which was not sufficient for making an exact diagnosis (34).

In a study of our group with 561 patients cared for on an MSU, the blood biomarker copeptin (C-terminal proAVP) showed no statistically significant differences in its levels in patients with cerebrovascular disease and other neurological diseases, as well as vascular and non-vascular diseases (35).

With the new approach of metabolomics, four biomarkers, asymmetrical and symmetrical dimethylarginine, pregnenolone sulfate, and adenosine, could differentiate 508 AIS from 349 SM patients, but further research is necessary to confirm these results (48).

Apart from the discrimination between AIS, ICH, and SM patients, further research questions will be investigated with the help of blood biomarkers. The Biomarkers of Acute Stroke Etiology (BASE) study, started in 2014, focuses on the determination of the etiology in patients with ischemic strokes or transient ischemic attacks (TIAs) by blood biomarkers (49).

In all studies investigating blood biomarkers in acute stroke care, two limitations were identified.

First, patients especially benefit from IVT and MT if these therapies are initiated very early after the onset of symptoms. Therefore, an early definitive diagnosis of AIS (and exclusion of ICB) by regular medical services with the help of POC diagnostics is desirable. However, most studies have investigated blood biomarkers beyond the time window for IVT; therefore, their use for a treatment decision-based approach cannot be evaluated (25). By drawing blood in the prehospital setting as soon as possible, earlier identification of concentrations of potential biomarkers is possible.

Second, one blood biomarker alone was not sufficient to distinguish between AIS, ICH, and SM with an acceptable degree of sensitivity, specificity, and positive and negative predictive values. Therefore, a “multi-omics” concept with the simultaneous detection of a sample of proteins (proteomics), genes (genomics), RNAs (transcriptomics), and metabolites (metabolomics) or a combination of them was proposed (50).

## Conclusions

Many blood biomarkers have been studied to distinguish AIS from ICH and SM patients, but not in the ultra-early prehospital phase after the onset of symptoms. However, the most important objective when deciding for or against IVT in suspected AIS patients is to rule out ICH, and to date, no single set of biomarkers has been identified to achieve this goal with acceptable certainty. In this study, we will use the state-of-the-art approaches in proteomics, transcriptomics, and metabolomics to find one or a set of new promising biomarkers to improve the differentiation between AIS and ICH patients. These approaches should also improve discrimination between stroke and SM patients. With two blood samples at different time points in the ultra-early phase after symptom onset, the discriminatory value of changing concentrations over time of these biomarkers will be investigated.

## Ethics statement

The studies involving human participants were reviewed and approved by Ethics Committee of the Charité - Universitätsmedizin Berlin. The patients/participants provided their written informed consent to participate in this study.

## Author contributions

HA, AK, JW, FG, and IL-M planned the study. FG, LB, CH-S, ED, EF, MZ, AK, CW, and JW included study participants during STEMO deployments as emergency physicians. ES and MW supervised the study at their respective study sites. LH and ML processed study samples on board STEMO. FG, LB, ED, CH-S, and MZ did follow-up surveys. LH, ML, and IL-M organized and ensured the quality of large parts of the administrative process of ordering, coding, labeling, packing, and transporting study samples. CD-H was responsible for certain administrative parts of the study at the BIH. FK wrote large parts of the statistical analysis part of the manuscript. IL-M organized large parts of the follow-up survey process. FG wrote the study protocol with repeated revisions from JW. KK, HA, and JW critically revised the content of the manuscript. LB and IL-M registered the study with the German Clinical Trials Register. All authors contributed to the article and approved the submitted version.
